# Thermal Stability, Optical Properties, and Gamma Shielding Properties of Tellurite Glass Modified with Potassium Chloride

**DOI:** 10.3390/ma15072403

**Published:** 2022-03-24

**Authors:** Khalid I. Hussein, Aref M. Al-Syadi, Mohammed S. Alqahtani, Nehal Elkhoshkhany, Hamed Algarni, Manuela Reben, El Sayed Yousef

**Affiliations:** 1 Department of Radiological Sciences, College of Applied Medical Sciences, King Khalid University, Abha 61421, Saudi Arabia; mosalqhtani@kku.edu.sa; 2 Department of Medical Physics and Instrumentation, National Cancer Institute, University of Gezira, Wad Medani 2667, Sudan; 3 Department of Physics, Faculty of Science and Arts, Najran University, Najran 11001, Saudi Arabia; arefalsyadi@yahoo.com; 4 Physics Department, Faculty of Education, Ibb University, Ibb 70270, Yemen; 5 BioImaging Unit, Space Research Centre, Department of Physics and Astronomy, University of Leicester, Leicester LE1 7RH, UK; 6 Physics Department, College of Arts and Sciences, Jouf University, Tabrjal 74713, Saudi Arabia; nmak2002@hotmail.com; 7 Department of Material Science, Institute of Graduate Studies and Researches, Alexandria University, 163 Horreya Avenue, Shatby, Alexandria 21526, Egypt; 8 Physics Department, Faculty of Science, King Khalid University, Abha 61413, Saudi Arabia; halgarni@hotmail.com (H.A.); omn_yousef2000@yahoo.com (E.S.Y.); 9 Research Center for Advanced Materials Science (RCAMS), King Khalid University, Abha 61413, Saudi Arabia; 10 Faculty of Materials Science and Ceramics, AGH—University of Science and Technology, al. Mickiewicza 30, 30-059 Krakow, Poland; manuelar@agh.edu.pl

**Keywords:** tellurite glasses, DTA, thermal characteristics, shielding parameters, potassium chloride, optical properties

## Abstract

The synthesized glass system with a composition of (80-x) TeO_2_-10P_2_O_5_-10Nb_2_O_5_-xKCl mol% (where x = 5, 10, 15, 20, and 25) was successfully fabricated. The density (ρ) and molar volume (V_m_) have been calculated. The investigated glasses were characterized using different analysis methods (differential thermal analysis (DTA) and UV-VIS-NIR spectroscopy). The radiation shielding effectiveness of the synthesized glass system was evaluated using different shielding parameters, such as mass and linear attenuation coefficients (MAC, LAC), half-value layer (HVL), mean free path (MFP), effective atomic number (Z_eff_), and effective electron number (N_eff_). The results showed that with the increasing potassium chloride (KCl) concentration and decreasing tellurium oxide (TeO_2_) concentration, the density, refractive index, Urbach energy (E_u_), and glass transition temperature (T_g_) decreased, while the optical energy gap (E_opt_) and thermal stability increased. As the KCl concentration increases, the values of MAC, LAC, and Z_eff_ increase in the following order: TPNK5 % > TPNK10 % > TPNK15 % > TPNK20 % > TPNK25 %. Additionally, the shielding effectiveness of TPNK glass system showed good performance compared with some standard materials. The synthesized glass with a minimum KCl content has both good shielding effectiveness and good optical properties, in addition to reasonable thermal stability, which makes it suitable for shielding and optical applications.

## 1. Introduction

Tellurite glasses have received increased attention due to their unique physical and chemical features, as compared with other oxide glasses, such as phosphates and silicates. In particular, tellurite glasses have high dielectric constant, excellent third-order nonlinear optical properties, low phonon energy, high refractive index, good infrared transmittance, low melting temperature, higher rare-earth ion solubility, and large thermo-optical coefficient [1,2,3,4].

Tellurite glasses are promising materials for a wide range of applications, including erasable optical recording media, optical switching devices, laser hosts, second harmonic generation, and Raman amplification due to their outstanding characteristics [2,3,5,6,7,8]. In the last few years, tellurite glasses have also received increased attention in both fundamental research and the production of optical devices. This is mainly due to the fact that tellurite-based optical glasses have good transparency in the VIS–infrared spectrum, as well as high corrosion resistance. Furthermore, tellurite glasses could be employed in the manufacture of fiber, planer broadband amplifiers, and lasers [9,10].

P_2_O_5_, B_2_O_3_, and other glass-forming oxides, such as alkali, rare-earth, transition, and post-transition metal ions, can be added to the host glass matrix to increase the melt-quenching and thermal stability, as well as optical capabilities. The glass system characteristics have improved significantly, making them ideal candidates for a wide range of optical and radiation detection applications [1,4,11,12,13,14,15]. When Nb_2_O_5_ is added to TeO_2_, the glass develops a narrow domain, improves vitrification, and raises the refractive index [16,17]. The addition of alkali ions to tellurite glass, such as Li, Na, and K, can reduce network connectivity and bond strengthening, as well as lower the melting point [18,19,20]. On the other hand, tellurium oxide (TeO_2_), as the principal but conditional glass-former, does not transition to the glassy state as a pure oxide under typical conditions due to the anomalous glass-forming behavior of tellurite glasses. As a result, the addition of alkali ions to tellurite-based glasses makes them more likely to form glass and creates non-bridging oxygen (NBO) sites, which lowers the average coordination number [20].

The structure of tellurite-based glasses is of interest due to the presence of two types of basic structural units, TeO_4_ trigonal bi-pyramid (tbp) and TeO_3_ trigonal pyramid (tp). Additionally, understanding the temperature stability against crystallization and structural relaxation behavior in the glass transition area is required to create tellurite-based glasses as new optical functional materials. However, this information is rare.

Furthermore, glasses are presently considered as one of the most effective materials for radiation shielding due to their multiple advantages over many other materials, such as high density, transparency to visible light, non-toxicity, and ease of manufacture [21,22,23,24,25,26,27,28,29]. Tellurite glasses, which are made of glass, have been shown to be excellent shielding materials for Gamma rays and neutrons [22,23,24,25,26,27,28]. As tellurium dioxide cannot form a stable glass system on its own, numerous modifiers have been studied to improve the structure and production of tellurite glasses. Al-Hadeethi et al. [22] investigated the physical characteristics of glass systems (Bi_2_O_3_-B_2_O_3_-TeO_2_-TiO_3_ and PbO-ZnO-TeO_2_-B_2_O_3_) in the diagnostic energy range at photon energies ranging from 30 to 80 kVp. Increases in the mole percentage of tellurium dioxide (TeO2) resulted in increased glass system density and attenuation coefficients. However, the transmission of X-ray photons and HVL was reduced, especially at photon energies of 70 and 80 keV. Hussein et al. [23] studied the shielding properties and glass production of four tellurite-based glasses doped with various metal oxides. The results revealed that of all the samples, the one containing barium oxide (BaO) had the highest mass and linear attenuation coefficients.

The addition of modifiers, such as alkali ions, can enhance the optical properties dramatically, especially if added to glasses that host heavy element oxides. The aim of this study is to investigate the Gamma shielding effectiveness, optical properties, and thermal stability of TeO_2_-P_2_O_5_-Nb_2_O_5_-KCl glass doped with heavy metal oxide, as well as perform an optical spectroscopic analysis on the system and investigate how these properties change with the addition of alkaline, such as KCl.

## 2. Materials and Methods

### 2.1. Sample Preparation

Using the conventional quench-melting method, glass systems with a composition of (80-x) TeO_2_-10P_2_O_5_-10Nb_2_O_5_-xKCl mol% (x = 5, 10, 15, 20, and 25) were synthesized. A specific weight of raw metal oxides (TeO_2_, P_2_O_5_, Nb_2_O_5_, and KCl from Sigma-Aldrich with purity ≥99%) was mixed and placed in a platinum crucible, which was heated to 950 °C for 30 min in a melting furnace, with the melt stirred continuously. The extremely viscous melt was cast in a graphite mold. Within the furnace, the quenched samples were annealed at 300 °C for 2 h prior to cooling to room temperature. Table 1 shows the names of the prepared glass samples: TPNK05 %, TPNK10 %, TPNK15 %, TPNK20 %, and TPNK25 %. A lapping machine with 600 grades and soft fine AlO_3_ powder was used to polish the prepared samples. To meet the optical measures, the opposite faces were polished optically flat and parallel. The densities of the as-synthesized glasses were measured with an helium pycnometer (UltraPyc1200e, Odelzhausen, Germany) with an accuracy of 0.0003 %. The following formula [25] was used to calculate the theoretical density (ρ) values of as-synthesized glasses:

(1) $$\rho =\frac{X_{{\mathrm{TeO}}_{2}}M_{{\mathrm{TeO}}_{2}}\rho_{{\mathrm{TeO}}_{2}}+X_{P_{2}O_{5}}M_{P_{2}O_{5}}\rho_{P_{2}O_{5}}+X_{{\mathrm{Nb}}_{2}O_{5}}M_{{\mathrm{Nb}}_{2}O_{5}}\rho_{{\mathrm{Nb}}_{2}O_{5}}+{\mathrm{xX}}_{\mathrm{KCl}}M_{\mathrm{KCl}}\rho_{\mathrm{KCl}}}{X_{{\mathrm{TeO}}_{2}}M_{{\mathrm{TeO}}_{2}}+X_{P_{2}O_{5}}M_{P_{2}O_{5}}+X_{{\mathrm{Nb}}_{2}O_{5}}M_{{\mathrm{Nb}}_{2}O_{5}}+X_{\mathrm{KCl}}M_{\mathrm{KCl}}}$$

where X_TeO2_, X_P2O5_, X_Nb2O5_, and X_KCl_ are the mole fraction of TeO_2_, P_2_O_5_, Nb_2_O_5_, and KCl, respectively. M_TeO2_, M_P2O5_, M_Nb2O5_, and M_KCl_ are the molecular weight of TeO_2_, P_2_O_5_, Nb_2_O_5_, and KCl (159.6, 283.9, 265.81, and 74.5513 g/mol), respectively. Additionally, ρ_TeO2_, ρ_P2O5_, ρ_Nb2O5_, and ρ_KCl_ are the densities of TeO_2_, P_2_O_5_, Nb_2_O_5_, and KCl (5.67, 2.39, 4.6, and 1.984 g/cm^3^), respectively.

### 2.2. Thermal Stability and Optical Properties

The glass transition temperature T_g_, onset crystallization temperature T_c_, and peak crystallization temperature T_p_ were obtained from the recorded thermograms using the Shimadzu differential thermal analyzer (DTA-50, Kyoto, Japan) in nitrogen medium, at a heating rate of 15 K/min over a range of 800 °C. Using the UV–VIS–NIR spectrophotometer (JASCO V-570, Tokyo, Japan), the optical absorption spectra were measured at wavelengths from 200 to 2500 nm.

The molar volume (V_m_) is calculated from the composition and density using the following Equation (2):

(2) $$V_{m}=\frac{M_{w}}{\rho }$$

where M_w_ is defined as the total molecular weight of sample composition.

The molar refractivity can be used to determine the overall polarizability of a mole of a material, which is used to investigate the role of ionic packing in influencing the refractive index of glass materials (R_m_). The following Equation can be used to calculate R_m_ [12,13]:

(3) $$R_{m}=\left(\frac{n^{2}-1}{n^{2}+2}\right)V_{m}$$

The molar polarizability of the glass (α_m_) is proportional to R_m_ and it was obtained by the following relationship [13]:

(4) $$\alpha_{m}=\left(\frac{3}{4{\pi N}_{A}}\right)R_{m}$$

where N_A_ is the Avogadro number. Table 2 shows the values of R_m_ and α_m_. As the concentration of KCl increases, the values also increase. To determine whether a substance is metallic or non-metallic, we use the following Equation (5):

(5) $$M=1-\frac{R_{m}}{V_{m}}$$

If R_m_/V_m_ < 1, (i.e., M > 0), the materials demonstrate an insulating nature. However, if R_m_/V_m_ > 1, (i.e., M < 0), the materials show a metallic nature.

### 2.3. Radiation Shielding Parameters

Glasses made from raw materials were tested for radiation shielding using different parameters, such as MAC, LAC, HVL, and MFP. The Z_eff_ and N_eff_ were also used to determine the effectiveness of the glass shielded materials. The linear attenuation coefficient (µ) can be used to quantify the interaction of Gamma rays with materials, which can be computed using the following Lambert–Beer rule [29]:

(6) $$\;I=I_{0}e^{-\mu x}$$

where I_0_, I, µ, and ρ are the initial photons, transmitted photons, and linear attenuation coefficient (${\mathrm{cm}}^{-1}$), respectively.

The mass attenuation coefficient may be calculated using the following equation [27,28,29]:

(7) $$\mu_{m}=\underset{i}{\sum }w_{i}{\left(\frac{\mu }{\rho }\right)}_{i}\;$$

where $w_{i}$ is the constituent element’s weight fraction, ${\left(\frac{\mu }{\rho }\right)}_{i}$ is the mass attenuation of the ith atomic element, and ρ is the glass density.

The half value layer, tenth value layer, and mean free path can be calculated using the following relations [30,31,32]:

(8) $$\mathrm{HVL}=\frac{0.693}{\mathrm{LAC}},\;\mathrm{TVL}=\frac{2.303}{\mathrm{LAC}}\;\mathrm{and}\;\mathrm{MFP}=\frac{1}{\mathrm{LAC}}$$

The effective atomic number ($Z_{\mathrm{eff}})$ can be estimated using the following Equation (9) [33]:

(9) $$Z_{\mathrm{eff}}=\frac{\sum {\mathrm{if}}_{i}A_{i}\left(\frac{\mu }{\rho }\right)i}{\sum j\frac{A_{j}}{Z_{j}}\left(\frac{\mu }{\rho }\right)j}$$

where $f_{i}$ is the ith atomic element of the mole fraction, $A_{i}$ is the atomic weight of the ith atomic element, and $Z_{j}$ is the atomic number.

Furthermore, the effective electron density ($N_{\mathrm{eff}}$) can be estimated using the following equation [33,34,35]:

(10) $$N_{\mathrm{eff}}=\frac{N_{A}}{M}Z_{\mathrm{eff}}\sum n_{i}$$

where $N_{A}$ is the Avogadro constant.

The shielding and optical parameters were calculated using the new developed software MIKE software (King Khalid University, Version 1, 2021, Abha, Saudi Arabia) [36].

## 3. Results and Discussion

### 3.1. Physical Parameters of the As-Synthesized Glasses

**Figure 1:**
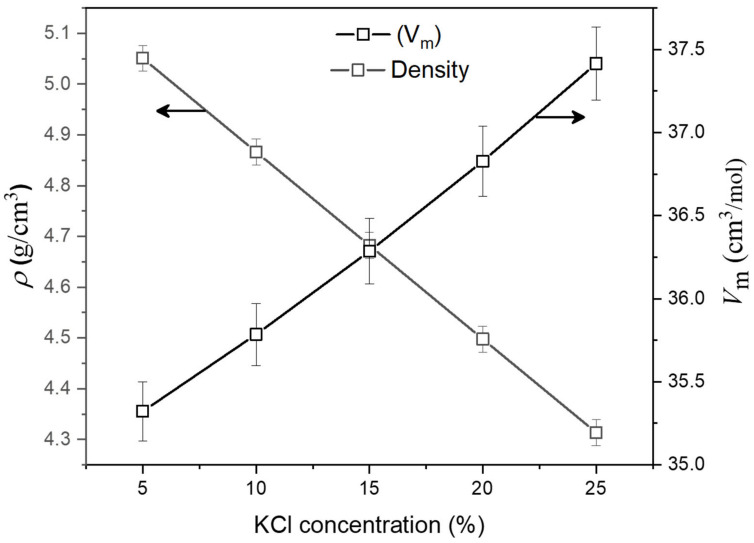
Dependence of glass density (ρ) and molar volume (V_m_) on the glass composition x.

The composition, density (ρ), and molar volume (V_m_) of the TPNK glass system, as well as the theoretical and measured values are shown in Table 1. The measured values were employed in the subsequent calculation for a more precise estimation of the characteristics of the prepared materials. The effect of KCl concentration on the density and molar volume of as-synthesized glasses is shown in Figure 1. The density of the glass decreases while the molar volume increases as the KCl concentration increases. This is due to KCl’s low molecular weight compared to TeO_2_.

### 3.2. Thermal Characteristics

**Figure 2:**
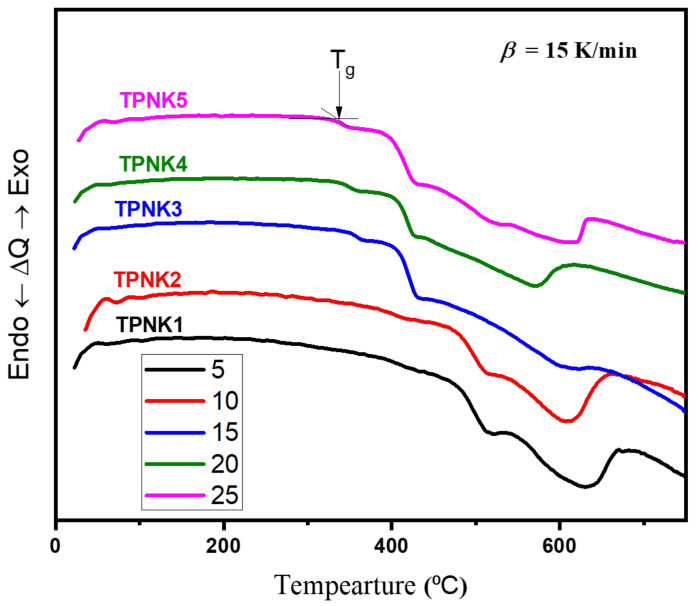
DTA profiles of TPNK glass system at a heating rate of 15 °C/min.

As illustrated in Figure 2, DTA curves at a heating rate (β) of 15 °C/min are used to depict the thermal behavior of TPNK glass samples. The glassy nature of the as-synthesized glasses is confirmed by the identical shapes of all the curves. Table 2 shows the glass transition temperature (T_g_), the onset of crystallization temperature (T_c_), and the peak crystallization temperature (T_p_).

The glass transition point is the first thermal property to be attained when the powder glass is heated, followed by the crystallization point, which is the glass’s transformation from an amorphous to crystalline state. Table 3 shows that as the amount of KCl in the solution increases, T_g_ drops. In general, T_g_ can be used to determine the rigidity of prepared glass samples [18,37]. Alkali ions, such as Li, Na, and K, can be added to tellurite glass to reduce network connectivity and reinforce bonds [18,19,20], which indicates that as the KCl concentration increases, T_g_ drops. On the other hand, T_g_ provides information on the glass network’s bond strength and connectivity. Moreover, T_g_ is known to increase as the connectivity and bond strength of the glass increase [38]. These T_g_ values are similar to those seen in tellurite-based glasses [39,40].

To help readers understand the effect of the addition of KCl to the TeO_2_ network, we will explain how it works. TeO_4_ trigonal bi-pyramids, in which one of the equatorial sites is occupied by a lone pair of electrons, and most of the tellurium atoms are bonded at their vertices by a lone pair of electrons, are the basic structural units of tellurite glasses with high TeO_2_ concentration (Te–O–Te linkage). When an alkali ion is added to tellurite glass, the Te–O_ax_ and Te–O_eq_ bonds weaken, and the TeO_4_ trigonal bi-pyramid network separates, resulting in the formation of NBO atoms in both the Te–O_eq_ and Te–O_ax_ links [41,42]. As a result, the change in KCl content produces a structural change in the coordination polyhedron in tellurite-based glasses with an alkali ion as a modifier. Tellurite glasses create both three and four coordination tellurium polyhedra at the same time [43]. The alkali ion modifier is responsible for the conversion of TeO_4_ polyhedra into TeO_3_ polyhedra. As the alkali ion modifier level rises, the ratio of TeO_3_ trigonal pyramid to TeO_4_ trigonal bi-pyramid also rises. The electron pairs of the extra TeO_3_ trigonal pyramidal groups do not interact well with each other [18].

As a preliminary estimate of the glass thermal stability, the thermal stability factor ∆T = (T_c_ − T_g_) was utilized. It is preferable to have a high T value to obtain an extensive operating range, such as during the fabrication process [44,45,46,47]. Sestak [48,49] investigated Hruby’s coefficient and glass compositional dependencies, which evolved into H = ∆T/T_g_. Table 2 shows the thermal stability factor (∆T) and Hruby’s coefficient (H), which are crucial in defining the glass devitrification process [18,37]. Glass samples in their as-prepared stage are a potential choice for large bulk glass and optical fiber production due to the considerable ΔT (ΔT > 100 °C) required in optical fiber construction to avoid crystallization during the fabrication process [13,37]. The following Equation can be used to determine KSP, which is a metric that shows how well the glass can prevent crystals from forming [13]:

(11) $$K_{\mathrm{SP}}=\frac{\left(T_{p}-T_{c}\right)\left(T_{p}-T_{g}\right)}{T_{g}}$$

Table 2 shows the K_SP_ values for the as-synthesized glasses, which are within the range of tellurite-based glasses, including alkali, alkaline, and heavy metal ions [13,50].

**Figure 3:**
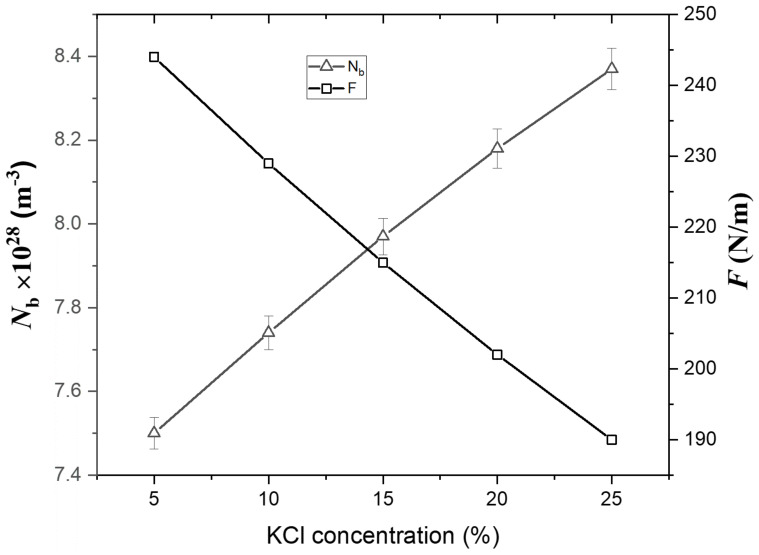
Dependence of the bonds per unit volume (N_b_) and the average bond stretching force constant (F) on the KCl content.

Using the following Equations [10,14,36,51], it is crucial to investigate the change in T_g_ as a function of the number of bonds per unit volume (Nb) and the average bond stretching force constant (F):

(12) $$T_{g}=f\left(N_{b},F\right),{\;\mathrm{where}\;N}_{b}=\frac{N_{A}}{V_{m}}\underset{i}{\sum }{\left(n_{f}x\right)}_{i}$$

(13) $$F=\underset{i}{\sum }{\left({\mathrm{xn}}_{f}f\right)}_{i}/\underset{i}{\sum }{\left({\mathrm{xn}}_{f}\right)}_{i}$$

(14) $$f=\frac{1.7}{r}$$

where n_f_ is the number of bonds per unit glass formula, N_A_ is the Avogadro number (N_A_ = 6.022 × 10^23^ mol^−1^), x_i_ is the mole fraction of the oxide, f is the first order stretching force constant, and r is the cation radius. The values of n_f_ and r for these oxides are reported in the literature [52,53,54,55,56,57]. The values of N_b_ and F for as-synthesized glasses are shown in Table 1. Figure 3 illustrates the dependence of N_b_ and F on the KCl content. It is observed that the value of N_b_ increases from 7.50 × 10^28^ to 8.37 × 10^28^ m^−3^, while F decreases from 244 to 190 N m^−1^ with an increase in KCl content from 5 to 25 mol%. Essentially, this implies a weaker interatomic interaction between the cation and oxygen due to the decreased F in these synthesized glasses’ structures. Moreover, the development of additional NBO made the glass network less dense, which led to less F.

### 3.3. Optical Properties

**Figure 4:**
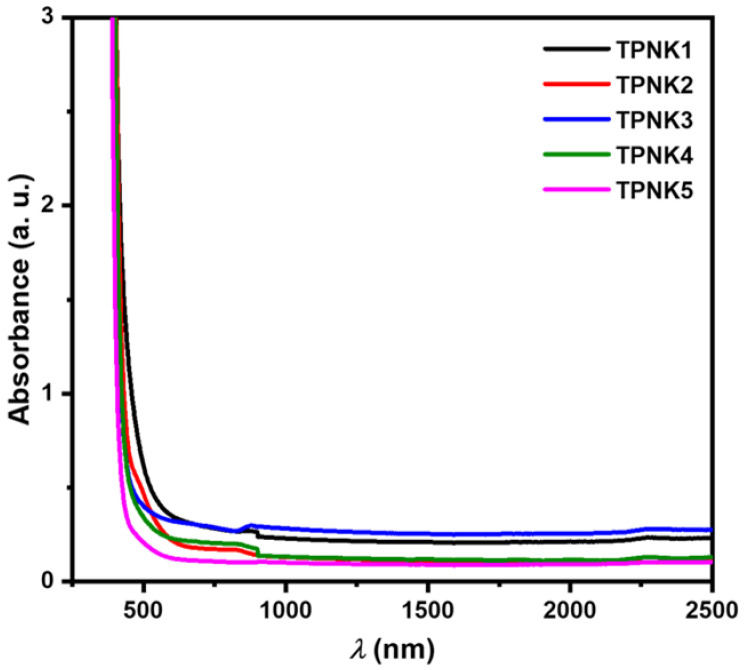
UV–VIS–NIR absorbance spectra of TPNK glass system.

Figure 4 shows the absorption spectra of the as-synthesized glasses in UV–VIS–NIR. Electron transfers from unexcited to excited states generate absorption in the UV–VIS–NIR spectral ranges. Figure 4 shows that the sharp edges were not present, which indicates that the as-synthesized samples were in the amorphous phase [12]. The following Equation (15) [12,14] can be used to compute the optical absorption coefficient (α):

(15) $$\alpha =\frac{1}{d}\ln\left(\frac{I_{0}}{I_{t}}\right)=2.303\frac{A}{d}$$

where d denotes the sample thickness, I_0_ and I_t_ denote the light intensity before and after passing through the sample, and A denotes the absorbance. Absorbance is communicated by the factor ln (I_0_/I_t_). In many amorphous semiconductors, the optical absorption coefficient (α) in the optical region at the band edge has an exponential dependency on photon energy (hv) and follows an empirical relationship proposed by Urbach [12,58]:

(16) $$\alpha =C\;\exp(\frac{h\nu }{E_{u}})$$

where C is a constant and Eu is the Urbach energy, which is related to the breadth of the band gap’s tail of localized states.

**Figure 5:**
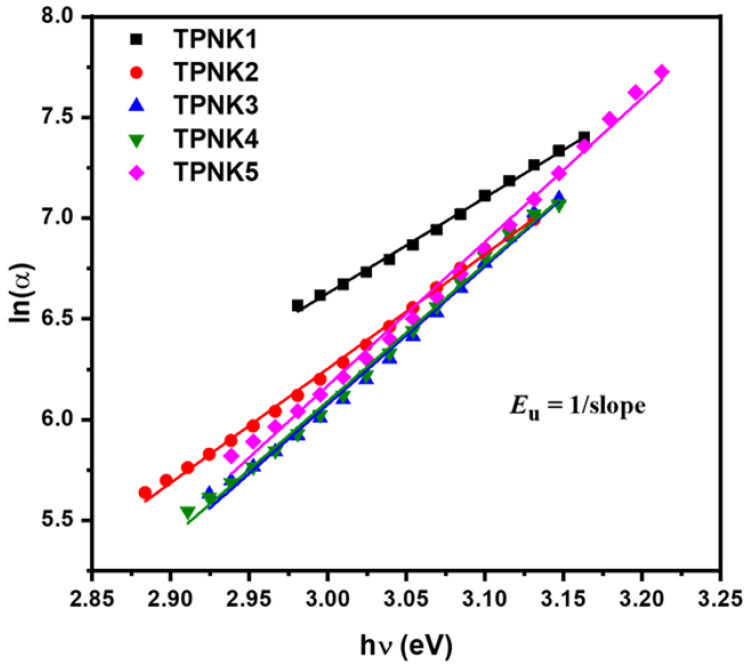
The ln (α) as a function of hν of TPNK glass system.

The phonon-assisted indirect electronic transitions are thought to be responsible for the E_u_ physical origin. The reciprocals of the slopes of the linear section of lnα vs. hv curves in the lower photon energy areas were used to calculate E_u_ values, as shown in Figure 5. Table 3 shows the E_u_ values for as-synthesized glasses. The large value of E_u_ indicated a high tendency for defects and a reduction in long-range order [13,59]. These glasses have Urbach energies, which are similar to those reported for other inorganic glasses [13]. With the increasing KCl content, the E_u_ value for as-synthesized glasses drops from 0.211 to 0.144 eV.

The addition of KCl loosens the packing of the glass samples by forming a Te–O–K bridge structure and increasing the number of NBO (i.e., TeO_4_ units are converted to TeO_3_ units, resulting in an increase in NBO), decreasing network connectivity and bond strengthening [18,19,20], and reducing E_u_.

The optical band gap (E_opt_) is determined using the Mott–Davis [60] relationship between the absorption coefficient (α) and the photon energy (hv) proposed for amorphous materials:

(17) $$\alpha \;h\nu =B{\left(h\nu -E_{\mathrm{opt}}\right)}^{s}$$

where s is a constant and B is a variable depending on the mechanism of interband transition.

**Figure 6:**
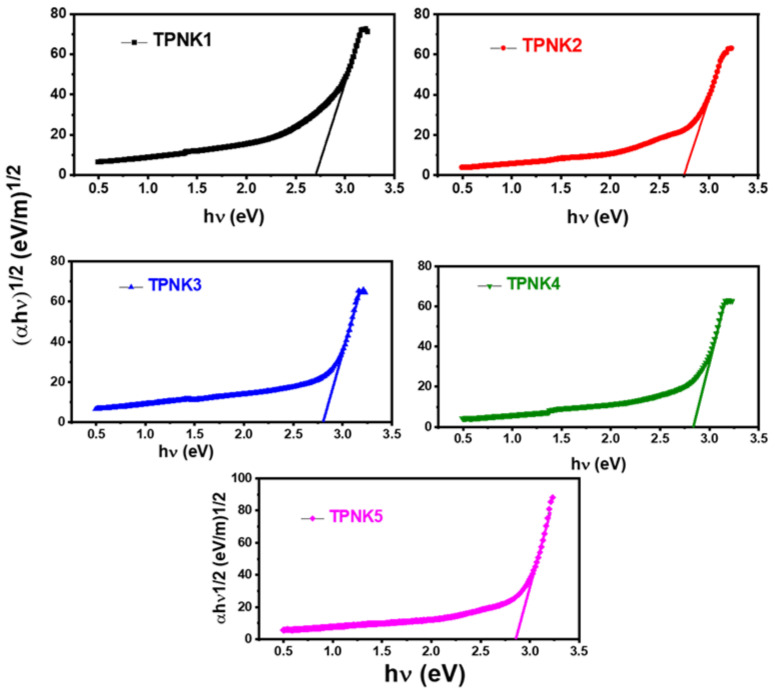
Tauc’s plot (αhν)^1/2^ vs. hν of TPNK glass system.

**Figure 7:**
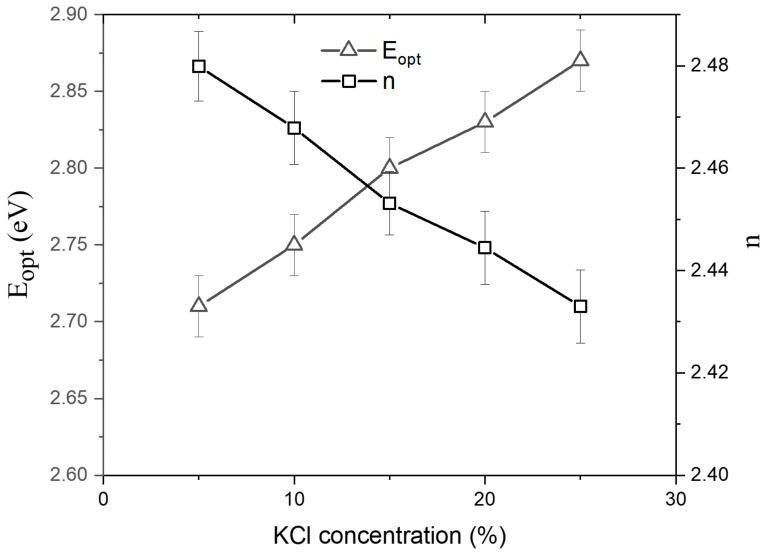
The variation of optical band gap (E_opt_) and leaner refractive index (n) as a function of the KCl content.

In most glasses, Equation (17) illustrates a straight line for s = 2 and is related to indirect allowed transitions. Tauc’s plot (hv)^1/2^ vs. (h) for as-synthesized glasses is shown in Figure 6. The E_opt_ of the present glasses was calculated by projecting the linear areas of the curves to meet the h axis at (h)^1/2^ = 0, and the results are shown in Table 3. As can be observed in Figure 7, the value of E_opt_ for as-synthesized glasses increases as the KCl concentration increases. The increase in E_opt_ can be attributed to the glass’s optical basicity [12,61]. The decrease in the effective electronic density of the valence shell of oxide ions is shown by an increase in optical basicity. As the network’s covalency decreases, the energy band gap increases. As a result, as the number of strong linkages in the glass network decreases, the band gap increases (as seen by a drop in glass transition temperature (T_g_)) [12]. On the other hand, as the KCl concentration increases, the glass samples become more loosely packed due to the increased NBO, reducing network connectivity and bond strengthening [18,19,20]. As a result, the T_g_ drops, while the E_opt_ increases.

The refractive index is the physical parameter of the glass material that should be determined in the fabrication of the optical devices. The refractive index (*n*) can be found from the E_opt_ values using the following Equation [62]:

(18) $$\frac{n^{2}-1}{n^{2}+2}=1-\sqrt{\frac{E_{\mathrm{opt}}}{20}}$$

The values of n are listed in Table 3. It is observed that the value of n for the as-synthesized glasses increases with an increase in the KCl content, as clearly seen in Figure 7. The refractive index of the as-synthesized glasses decreased as the KCl content increased (i.e., with the decreased TeO_2_ content). This is due to a decrease in density.

The values of R_m_ and α_m_ are shown in Table 3. These values increase with an increase in KCl content. The metallization criterion (M) provides us with information regarding the metallic or non-metallic nature of solids, which depends on the ratio of molar refractivity to molar volume (R_m_/V_m_). If R_m_/V_m_ < 1, (i.e., M > 0), the materials demonstrate an insulating nature. However, if R_m_/V_m_ > 1, (i.e., M < 0), the materials show a metallic nature. Table 3 lists the metallization creation values in the range of 0.368–0.379. Glasses made from this material showed an insulating property [13,63].

### 3.4. Radiation Shielding Properties

**Figure 8:**
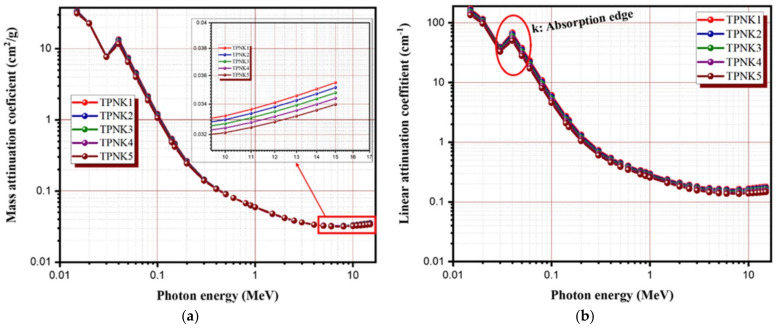
Radiation shielding parameters of TPNK system. (**a**) MAC; (**b**) LAC.

The shielding efficiency of the as-synthesized glasses was studied at a wide energy ranging between 0.15 and 15 MeV. The radiation parameters were calculated using the software MIKE. Figure 8a,b shows the mass and linear attenuation coefficients of the TPNK system. As shown in Figure 8a,b, the values of MAC and LAC decrease sharply due to the photoelectric absorption process, which is predominant in this energy range. Then, the curve gradually decreases as the energy increases due to the Compton effect and pair production process for energy above 50 keV. The k-absorption edge was recorded at an energy of 40 keV, which greatly influences the shielding efficiency of as-synthesized glasses at this energy range. As illustrated in Figure 8a,b, the values of MAC and LAC decrease as the KCl concentration increases, at the expense of a decrease in TeO_2_ concentration. Evidently, the sample coded TPNK1 has the highest values of MAC and LAC due to the high concentration of the heaviest metal oxide (TeO_2_) among the other samples. This outcome is in line with the findings of the previous study [22,23,24,25,26,27,28]. This result indicates that the optimum concentration of KCl is 5 mol%. This preserves tremendous optical and physical properties, while possessing good shielding performance.

**Figure 9:**
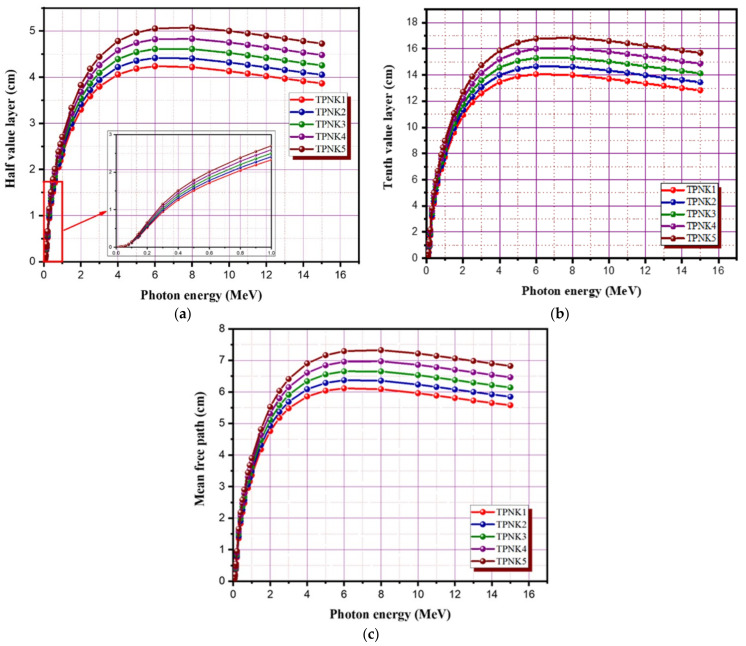
Radiation shielding parameters of TPNK system. (**a**) HVL; (**b**) TVL; (**c**) MFP.

**Figure 10:**
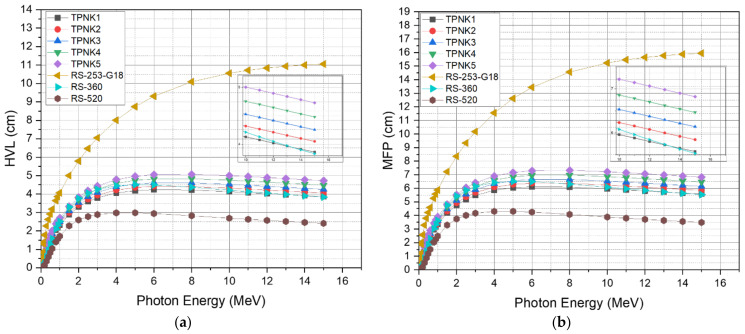
Shielding parameters of TPNK system compared with the standard materials. (**a**) HVL; (**b**) MPF.

The half-value layer and tenth-value layer denote the absorbance thickness required to reduce the photon flow by a half and a tenth, respectively. The variations in the HVL and TVL of the prepared glass samples with energies are seen in Figure 9a,b. The average distance between photon collisions with particles in a medium, in which a photon moves, is known as the mean free path (MFP). The values of HVL, TVL, and MFP demonstrate the shielding capability of the shielding glass material against Gamma radiation. As shown in Figure 9a–c, the values of HVL, TVL, and MFP increase with the increasing photon energy up to an energy of 6 MeV, beyond which the parameter values drop slightly. The maximum values of HVL, TVL, and MFP for sample TPNK1 are 4.24, 14.06, and 6.11, respectively, at 6 MeV. The recorded values of HVL, TVL, and MFP for all energies are in the order of TPNK1< TPNK2 < TPNK3 < TPNK4 < TPNK5. Sample TPNK1 shows the best shielding efficiency among the other samples. This result is consistent with the findings of MAC and LAC. This indicates that as the KCL concentration increases, the values of HVL, TVL, and MFP decrease, which results in better shielding efficiency. Furthermore, as shown in Figure 10a,b, the values of MFP and HVL of the investigated glasses were compared with those of commercially available shielding glass materials, namely RS-253-G18, RS-360, and RS-520 [64]. The RS-253-G18, RS-360, and RS-520 glass materials are commonly used due to their considerable shielding efficiency. Due to their high content of PbO (45% and 71%, respectively), RS-360 and RS-520 are more efficient than other radiation shielding glasses. For all energies, the prepared glass materials show a better shielding efficiency compared with the standard materials RS-360 and RS-253-G18, while RS-520 is slightly better compared with the prepared samples due to the high content of lead oxide in RS-520 glass.

**Figure 11:**
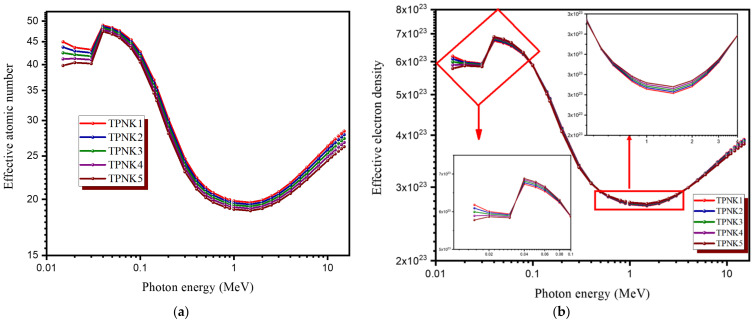
The radiation shielding parameters. (**a**) Effective atomic number (Z_eff_); (**b**) effective electron number (N_eff_).

To investigate the efficiency of the prepared glasses as shielding materials, the Z_eff_ and N_eff_ were also computed. Figure 11a,b shows how Z_eff_ and N_eff_ change as the photon energy and chemical composition change. With changing the photon energy and the chemical composition, both Z_eff_ and N_eff_ can significantly change. In the low photon region, Z_eff_ and N_eff_ values are high, whereas in the high photon region, they are low. This is due to partial photon processes that are proportional to the constituent elements’ atomic numbers (Z). The photoelectric process is Z^4^ dependent at low energy, whereas the Compton process is Z dependent. In reality, greater Z_eff_ values indicate better shielding performance. For instance, high-Z_eff_ materials have a greater chance of interacting with the Gamma ray. with high Z_eff_ materials, decreasing the photon energy to the point where it can no longer permeate the material. Furthermore, there are peaks in the Z_eff_ and N_eff_ curves around the photon energy of 0.0392 MeV due to the absorption edge of the Te element. As seen in Figure 11b, there are some differences in N_eff_ due to photon energies. The fluctuation in N_eff_ is due to the fact that it is proportional to the effective atomic number of the shielding material and inversely proportional to the mean atomic mass. At higher energies, a sample with the largest mean atomic mass will have slightly lower effective electron numbers (N_eff_). Figure 11b shows that TPNK1 glass has the highest N_eff_ value up to 0.03 MeV, while TPNK5 glass has the highest value above 0.04 MeV. Keeping the highest concentration of TeO_2_ and the lowest concentration of KCl will ensure that both the promising shielding efficiency and the strong thermal stability and good optical characteristics can be maintained.

## 4. Conclusions

The synthesized glasses (80-x) TeO_2_-10P_2_O_5_-10Nb_2_O_5_-xKCl mol% (where x = 5, 10, 15, 20, and 25) have been successfully synthesized by the melt-quenching technique. In this TPNK glass system, the increasing KCl concentration leads to a decrease in the glass transition temperature T_g_. The density of the glass samples decreased with the increasing KCl concentration due to its smaller atomic mass compared with TeO_2_, while the molar volume increased. The values of the optical energy gap (E_opt_), molar refractivity, and molar polarizability increased with the increasing KCl concentration. However, the Urbach energy (E_u_) and linear refractive index (n) decreased. The TPNK glass system insulating properties can be seen in the metallization creation values. The current glass system presents a high refractive index and high thermal stability. The shielding evaluation of the prepared glasses shows good performance compared with commercial standard materials. Moreover, TPNK1 shows the best performance among the investigated shielding materials due to the high concentration of TeO_2_. In conclusion, maintaining the maximum concentration of TeO_2_, while at the same time maintaining the minimum possible concentration of KCl will enable us to preserve both the promising shielding effectiveness, as well as the good thermal stability and good optical properties. This makes it an appropriate candidate to fabricate large bulk glass for shielding and optical applications.

## Figures and Tables

**Table 1:** Theoretical and measured density (ρ), molar volume (V_m_), number of bond per unit volume (N_b_), and average bond stretching force constant (F) of TPNK glass system.

Sample Code	Composition (mol%)	Theoretical ρ (g/cm^3^)	Experimental ρ (g/cm^3^)	V_m_ (cm^3^/mol)	N_b_ × 10^28^ (m^−3^)	F N/m
TPNK1	75TeO_2_-10P_2_O_5_-10Nb_2_O_5_-5KCl	4.9125	5.0507 ± 0.054	35.322	7.50	244
TPNK2	70TeO_2_-10P_2_O_5_-10Nb_2_O_5_-10KCl	4.8152	4.8664 ± 0.055	35.785	7.74	229
TPNK3	65TeO_2_-10P_2_O_5_-10Nb_2_O_5_-15KCl	4.7129	4.6821 ± 0.055	36.286	7.97	215
TPNK4	60TeO_2_-10P_2_O_5_-10Nb_2_O_5_-20KCl	4.6055	4.4978 ± 0.054	36.827	8.18	202
TPNK5	55TeO_2_-10P_2_O_5_-10Nb_2_O_5_-25KCl	4.4923	4.3135 ± 0.055	37.415	8.37	190

**Table 2:** Urbach energy (E_u_), indirect optical band gap (E_opt_), leaner refractive index (n), molar refractivity (R_m_), molar polarizability (α_m_), and metallization criterion (M) of TPNK glass system.

Sample Code	E_u_ (eV)	E_opt_ (eV)	N	R_m_ (Mol^−1^)	α_m_ (Å^−3^)	M
TPNK1	0.211	2.71	2.48	22.32	8.85	0.368
TPNK2	0.177	2.75	2.47	22.52	8.93	0.371
TPNK3	0.147	2.80	2.457	22.71	9.01	0.374
TPNK4	0.146	2.83	2.44	22.97	9.11	0.376
TPNK5	0.144	2.87	2.43	23.24	9.22	0.379

**Table 3:** The glass transformation temperature (T_g_), onset crystallization temperatures (T_c_), peak crystallization temperature (T_p_), thermal stability factor (∆T), Hruby’s coefficient (H), and parameter (K_SP_) of TPNK glass system.

Sample Code	T_g_ (°C)	T_c_ (°C)	T_p_ (°C)	∆T (°C)	H	K_SP_ (°C)
TPNK1	413	636	671	223	0.54	21.86
TPNK2	402	615	660	213	0.53	28.88
TPNK3	357	603	642	246	0.69	31.13
TPNK4	345	575	616	230	0.67	32.21
TPNK5	338	620	634	282	0.83	12.26

## Data Availability

Not applicable.
